# COVID-19: Are We Facing Secondary Pellagra Which Cannot Simply Be Cured by Vitamin B3?

**DOI:** 10.3390/ijms23084309

**Published:** 2022-04-13

**Authors:** Renata Novak Kujundžić

**Affiliations:** Laboratory for Epigenomics, Division of Molecular Medicine, Ruđer Bošković Institute, 10000 Zagreb, Croatia; rnovak@irb.hr; Tel.: +385-1-456-0949

**Keywords:** nicotinamide adenine dinucleotide, NAD^+^ salvage pathway, nicotinamide N-methyltransferase, aldehyde oxidase, obesity, diabetes, oxytocin, smell, taste, inflammaging

## Abstract

Immune response to SARS-CoV-2 and ensuing inflammation pose a huge challenge to the host’s nicotinamide adenine dinucleotide (NAD^+^) metabolism. Humans depend on vitamin B3 for biosynthesis of NAD^+^, indispensable for many metabolic and NAD^+^-consuming signaling reactions. The balance between its utilization and resynthesis is vitally important. Many extra-pulmonary symptoms of COVID-19 strikingly resemble those of pellagra, vitamin B3 deficiency (e.g., diarrhoea, dermatitis, oral cavity and tongue manifestations, loss of smell and taste, mental confusion). In most developed countries, pellagra is successfully eradicated by vitamin B3 fortification programs. Thus, conceivably, it has not been suspected as a cause of COVID-19 symptoms. Here, the deregulation of the NAD^+^ metabolism in response to the SARS-CoV-2 infection is reviewed, with special emphasis on the differences in the NAD^+^ biosynthetic pathway’s efficiency in conditions predisposing for the development of serious COVID-19. SARS-CoV-2 infection-induced NAD^+^ depletion and the elevated levels of its metabolites contribute to the development of a systemic disease. Acute liberation of nicotinamide (NAM) in antiviral NAD^+^-consuming reactions potentiates “NAM drain”, cooperatively mediated by nicotinamide N-methyltransferase and aldehyde oxidase. “NAM drain” compromises the NAD^+^ salvage pathway’s fail-safe function. The robustness of the host’s NAD^+^ salvage pathway, prior to the SARS-CoV-2 infection, is an important determinant of COVID-19 severity and persistence of certain symptoms upon resolution of infection.

## 1. Introduction

The coronavirus disease 2019 (COVID-19), caused by severe acute respiratory syndrome coronavirus 2 (SARS-CoV-2) infection, continues to pose an enormous global threat. This virus was first detected in Wuhan, China, in December of 2019. In January 2020, the first cases were detected outside of China and World Health Organization (WHO) declared a pandemic in March 2020 (http://who.int/, accessed on 2 March 2022) [1]. At the time of writing this paper, according to the WHO, there have been more than 435,000,000 confirmed COVID-19 cases and close to 6,000,000 deaths (http://who.int/, accessed on 2 March 2022).

Clinicians and scientists are making great efforts to understand this disease, its different clinical presentation and above all to understand why some, frequently observed, factors predispose people for developing serious/fatal disease. A lot has been learned, in the last two years, about the structure and biology of SARS-CoV-2, along with the changes it causes in the course of the disease progression. This information is invaluable since the pathogenesis of COVID-19 is closely related to the host’s defense responses to virus, inevitably affecting the host’s metabolism.

People infected with SARS-CoV-2 have a wide range of symptoms, from mild to those of serious illness. Although COVID-19 is primarily a respiratory disease, various extra-pulmonary symptoms, including gastrointestinal, neurologic, dermatological, renal, hepatic, cardiac, endocrine, thromboembolic, ocular and auditory, have been reported [2,3,4,5,6,7].

The most prominent changes, as a part of the immune response to SARS-CoV-2 infection, occur in the metabolism of nicotinamide adenine dinucleotide (NAD^+^), an essential cofactor in various cellular metabolic pathways [8,9,10,11,12,13]. Apart from the essential role of NAD^+^ and its reduced form (NADH) in various electron exchange-dependent biochemical reactions, NAD^+^ is continuously degraded by NAD^+^-consuming enzymes poly(ADP-ribose) polymerases (PARPs), sirtuins (SIRTs) and CD38. A byproduct of reactions catalyzed by NAD^+^-consuming enzymes is nicotinamide (NAM), which needs to be adequately recycled back to NAD^+^, in a salvage pathway, to sustain the NAD^+^ level [14,15]. During aging and in various pathological conditions, the fidelity of NAD^+^ replenishment upon its elevated consumption is sub-optimal, resulting in NAD^+^ level decline.

Predisposing conditions for developing serious/life-threatening disease are advanced age, obesity, diabetes, hypertension, cardiovascular and renal diseases, all related to disturbed NAD^+^ metabolism [16,17]. There is a possibility that the “fitness” of the host’s NAD^+^ metabolism to respond to the SARS-CoV-2 infection is at least partly accountable for various clinical manifestations of COVID-19.

Changes in the NAD^+^ metabolism, observed in COVID-19, may reflect endogenous vitamin B3 (also known as niacin, the generic name for nicotinic acid, nicotinamide and related derivatives, such as nicotinamide riboside) deficiency. This deficiency, independent of vitamin B3 dietary intake, may contribute to the development of pellagra-like symptoms. In the clinical practice, such symptoms have not been suspected to relate to vitamin B3 deficiency, historically linked to poverty and malnutrition [18,19]. However, pellagra-like COVID-19 symptoms are not restricted to the poorest people. On the contrary, COVID-19 equally takes its toll in the most developed societies, burdened by their high standard of living-mediated, metabolism-linked morbidities and high proportion of elderly population [1].

The possible mechanisms underlying the development of endogenous vitamin B3 deficiency, which may be further aggravated upon SARS-CoV-2 infection to result in different clinical manifestations of COVID-19, call for detailed scientific and clinical scrutiny.

## 2. NAD^+^ Biosynthesis with Some Sex- and Species-Related Differences

**Figure 1:**
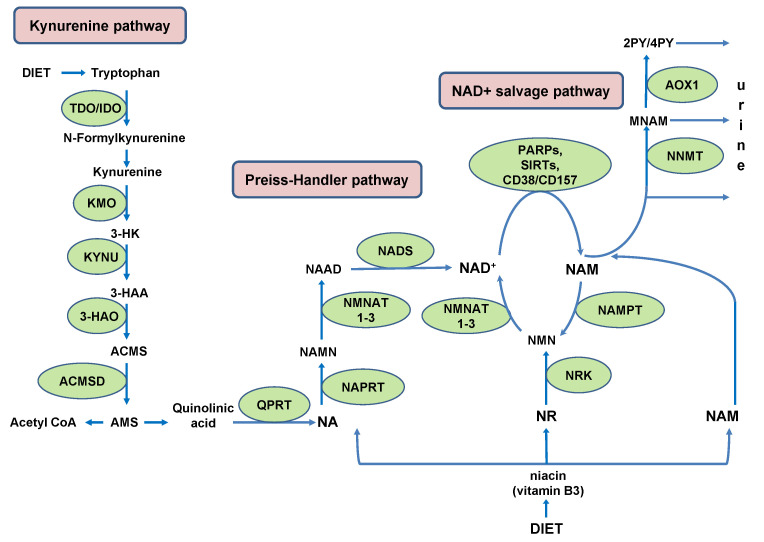
Nicotinamide adenine dinucleotide (NAD^+^) biosynthetic pathways. NAD^+^ is *de novo* synthesized from tryptophan in the kynurenine pathway. The rate-limiting enzymes tryptophan 2,3-dioxygenase (TDO) or indoleamine 2,3-dioxygenase (IDO) convert tryptophan to N-formylkynurenine, which is then transformed into L-kynurenine and converted to 3-hydroxykynurenine (3-HK) by kynurenine 3-monooxygenase (KMO). Kynureninase (KYNU) converts 3-HK to 3-hydroxyanthranilic acid (3-HAA), from which 3-hydroxyanthranilic acid oxygenase (3HAO) generates α-amino-β-carboxymuconate ε-semialdehyde (ACMS). ACMS is either diverted, by the activity of alpha-amino-beta-carboxy-muconatesemialdehyde decarboxylase (ACMSD), away from NAD^+^ synthesis or can spontaneously convert into qunilinic acid (QA). Qunolinate phosphoribosyltransferase (QPRT) transforms QA into nicotinamide mononucleotide (NAMN), at which point the kynurenine pathway converges with the Preiss-Handler pathway. In the Preiss-Handler pathway, nicotinic acid (NA) is converted by nicotinic acid phosphoribosyltransferase (NAPRT) to NAMN, which is transformed by nicotinamide mononucleotide adenylyltransferases (NMNAT 1–3) into nicotinic acid adenine dinucleotide (NAAD). Finally, NAD^+^ synthetase converts NAAD to NAD^+^. NAM liberated in NAD^+^-consuming reactions, catalyzed by PARPs, SIRTs and CD38/CD157, is recycled to NAD^+^ in the salvage pathway. Intracellular nicotinamide phosphoribosyltransferase (NAMPT) converts NAM to NMN, which is then converted to NAD^+^. In conditions of either excessive liberation of NAM in NAD^+^-consuming reactions, or dietary surplus, nicotinamide N-methyltransferase (NNMT) methylates NAM to generate N1-methylnicotinamide (MNAM) and prevent NAM’s inhibitory effect on PARPs and SIRTs. Aldehyde oxidase (AOX1) transforms MNAM into N1-methyl-2-pyridone-5-carboxamide (2PY) and N1-methyl-4-pyridone-3-carboxamide (4PY). NAM, MNAM, 2PY and 4PY are secreted from cells and excreted in urine.

In humans, NAD^+^ can be *de novo* synthesized from the essential amino acid tryptophan (Trp) in kynurenine pathway (KP). Under physiological conditions, the main site of *de novo* NAD^+^ synthesis from Trp is the liver, where tryptophan 2,3-dioxygenase (TDO) catalyzes rate-limiting conversion of Trp to N-formylkinurenine. Humans utilize Trp for NAD^+^ synthesis in KP with low efficiency (~60–70 mg Trp is utilized to generate an equivalent amount of NAD^+^ produced from 1 mg niacin) [20]. This low efficiency stems from high activity of human alpha-amino-beta-carboxy-muconatesemialdehyde decarboxylase (ACMSD). An intermediate Trp metabolite, alpha-amino-beta-carboxy-muconatesemialdehyde (ACMS), can either undergo spontaneous cyclization to qunolinic acid (QA), a precursor for NAD^+^ synthesis, or it can be decarboxylated by ACMSD to alpha-amino-muconate-semialdehyde (AMS). This is a limiting step which diverts ACMS from *de novo* NAD^+^ synthesis and channels it to the synthesis of acetyl coenzyme A (acetyl-CoA) (Figure 1) [21,22].

Through the generation of acetyl-CoA, ACMSD contributes to energy production in tricarboxylic acid (TCA) cycle. Accordingly, its expression is induced by energy deficiency during fasting and is suppressed by insulin upon feeding. It has been shown that *Acmsd* transcription is cooperatively regulated by hepatocyte nuclear factor 4 alpha (HNF4α) and peroxisome proliferator-activated receptor-γ coactivator 1-α (PGC1α) [23].

There are differences among species in the ACMSD activity. Palzer et al., have reported that higher activity of ACMSD in humans is responsible for their greater sensitivity to niacin depletion, when compared to mice. Mice, due to lower ACMSD activity, utilize Trp for NAD^+^ synthesis more efficiently than humans. Overexpression of ACMSD in mice makes them dependent on dietary niacin for NAD^+^ synthesis, comparable to humans. When fed a niacin-deficient diet, mice with overexpressed ACMSD develop NAD^+^ deficiency. This difference between man and mice should be considered when using mice as an animal model for studying NAD^+^ deficiency-associated human pathologies, as well as aging [21]. In addition to species differences, differences between sexes should be taken into account.

Shibata and Toda have investigated the reason for approximately two-fold higher mortality in females, compared to males, when affected by dietary insufficiency-caused pellagra. They have observed, in rats, that a diet containing estrone or testosterone had no effect on Trp level. However, an estrone-containing diet was associated with a lower conversion rate of Trp to niacin and a decrease in urinary excretion of NAM and its metabolites: N1-methylnicotinamide (MNAM), N1-methyl-2-pyridone-5-carboxamide (2PY) and N1-methyl-4-pyridone-3-carboxamide (4PY). Consistent with this observation, the activity of ACMSD was considerably higher in estrone-containing diet-fed rats compared to control rats (2.28 ± 0.17 vs. 0.81 ± 0.08 µmol/h/g of liver). Feeding rats with a testosterone-containing diet had no effect on the level of any of those parameters. Based on these experiments, the authors concluded that higher pellagra mortality in women is a consequence of decreased synthesis of NAD^+^ from Trp. As female sex hormones have the capacity to inhibit the synthesis of niacin from Trp, women of the reproductive age, and particularly during pregnancy, are at a higher risk of developing pellagra than men [24].

Tryptophan is mostly metabolized to NAM in the liver and released into the serum to be used by peripheral cells for NAD^+^ synthesis in the salvage pathway [25].

Outside the liver, most cells do not express all enzymes of the KP needed to convert Trp into NAD^+^. In extrahepatic tissues, KP is initiated by indoleamine 2,3-dioxygenase (IDO) [26]. Unlike TDO which has a housekeeping role in maintaining the homeostatic level of Trp, IDO is highly induced by various inflammatory stimuli [27]. It has been shown that resting macrophages are able to synthesize NAD^+^ from Trp, through completely executed KP, to maintain mitochondrial respiration, mandatory for an anti-inflammatory state with high phagocytic capability. However, upon innate immune challenge and during aging, KP is activated, but incompletely executed due to the suppressed activity of quinolinate phosphoribosyltransferase (QPRT) and its inability to metabolize QA further to NAD^+^. This is associated with NAD^+^ decline and progression of innate immune dysfunction during aging, contributing to the development of age-associated diseases [28].

In the serum of COVID-19 patients, a decreased level of Trp, accompanied by the accumulation of its metabolites kynurenine (KYN) and QA, likely represent a largely futile attempt of *de novo* NAD^+^ synthesis through highly upregulated KP [9,10,12,13].

In addition to its role in NAD^+^ synthesis, this metabolic pathway drives immune tolerance to restrain inflammation, but may delay virus clearance. The activation of immunosuppressive IDO1, estimated by KYN/Trp ratio in serum, is highly upregulated in critically ill COVID-19 patients. Elevated IDO1 negatively affects effector T cells, while increasing the proportion of T regulatory cells (Treg), correlating with the extent of lymphopenia and the severity of the disease [12].

Due to the low efficiency of Trp conversion to NAD^+^, the main route of NAD^+^ synthesis, in humans, is a salvage pathway from NAM, either obtained from dietary sources or liberated in NAD^+^-consuming reactions.

Considering the high rate of NAD^+^ degradation in NAD^+^-consuming reactions, its undisturbed recycling into NAD^+^ in the salvage pathway is of the utmost importance for maintaining physiological functions. Recently, Bockwoldt et al., have demonstrated that efficient recycling of NAD^+^ from NAM is based on two crucial evolutionary events: (1) structural adaptation of nicotinamide phosphoribosyltransferase (NAMPT), a key enzyme in the NAD^+^ salvage pathway, conferring it high affinity towards NAM; and (2) the occurrence of nicotinamide N-methyltransferase (NNMT), which by methylating NAM converts it into MNAM, facilitates its excretion and prevents its accumulation in the cell, where it would inhibit NAD^+^-dependent signaling reactions. Much higher NAMPT’s affinity towards NAM (*K_M_* 5 nM) than NNMT (*K_M_* 400 μM) implies that NNMT would be activated only under circumstances of a high increase in the NAM level. Based on calculations, it has been predicted that NAD^+^ consumption fluxes are higher, rather than lower, in the presence of NNMT. Those higher NAD^+^ consumption fluxes, in the presence of NNMT, however, decrease the steady-state concentration of NAD^+^. When NAD^+^ level exceedingly drops due to high NNMT activity, NAD^+^ consumption declines, limiting NAD^+^ salvage from NAM and closing a vicious cycle [29]. Not considered in those calculations is the presence of alternative inhibitors of NAD^+^-consuming reactions, in addition to NAM. The beneficial role of NNMT, commonly regarded as a “vitamin B3 clearance enzyme”, in preventing NAM-mediated inhibition of NAD^+^-consuming reactions can be jeopardized by the activity of aldehyde oxidase (AOX1). 

This enzyme metabolizes NNMT’s product MNAM into 2PY and 4PY [30]. In addition to being excreted, those metabolites inhibit PARP1 [31]. In the context of NNMT’s regulatory function in the NAD^+^ metabolism, the product inhibition of NNMT by MNAM should also be taken into account [32,33]. When metabolized by AOX1 into 2PY and 4PY, MNAM no longer engages in negative feedback regulation of NNMT activity. Unrestrained NNMT activity, in the presence of AOX1, whose expression is regulated by NRF2 in response to oxidative stress, together with decrease in NAM liberation due to the inhibition of PARP by 2PY/4PY, creates “NAM drain” and stalls NAD^+^ synthesis in the salvage pathway [34].

In humans, high dependency on NAM for NAD^+^ synthesis confers vulnerability to its deficiency-mediated decline in NAD^+^ levels, particularly associated with age-related metabolic changes. It has been estimated that up to 15–20% of the general population and 26% of elderly people may be vitamin B3 deficient, regardless of the B3 dietary intake [21].

During aging, human plasma concentration of 2PY increases. When comparing healthy young subjects (5–16 years old) to older healthy subjects (50–90 years old), Slominska et al., have found approximately a 2.6-fold higher plasma level of 2PY in older people. They have proposed that this increase in plasma concentration of 2PY is either a consequence of higher degradation of NAD^+^ or a decrease in NAM recycling into NAD^+^ [35].

In addition to age, AOX is regulated by sex hormones, positively by testosterone and negatively by estrogens, as shown in mice [36]. Teo et al., have recently reported that *in vitro*, in estrogen receptor-positive (ER+) human breast carcinoma cells MCF7, the physiological levels of estrogen suppress AOX1 expression [37].

With respect to MNAM metabolizing activity of AOX, humans and mice significantly differ. Whereas there is only one isoform of AOX in humans (AOX1), mice express four isoforms (AOX1-4), in a tissue-specific manner. In humans, AOX1 is most abundantly expressed in adrenal glands, adipose tissue and the liver, followed by trachea, glandular epithelium of the prostate, bone, kidney and connective tissue. In mice, only AOX2 and AOX3 can metabolize MNAM. Mouse AOX2 has much higher catalytic efficiency for MNAM (*k_cat_*/*K_M_* = 5.2 min^−1^µM^−1^) than AOX3 (*k_cat_*/*K_M_* = 0.7 min^−1^µM^−1^) [38]. The expression of AOX2 is highly restricted to the Bowman’s gland in the nasal cavity, while AOX3 is expressed in the liver, lung, oviduct and testis [39]. The tissue-specific expression of AOX isoforms, and their isoform-specific ability to use MNAM as a substrate, should be considered when using mice as an experimental model for investigating the involvement of AOX in the regulation of the NAD^+^ metabolism.

**Figure 2:**
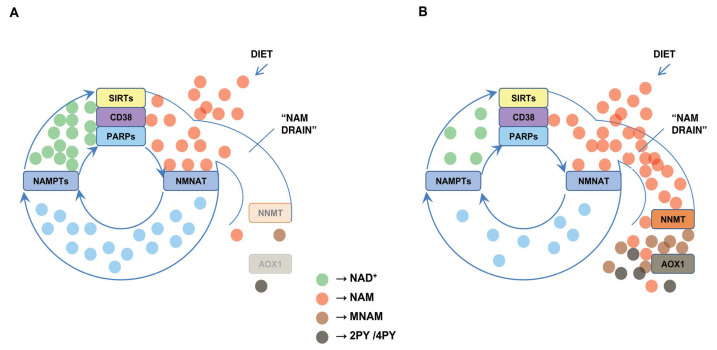
“NAM drain”-mediated NAD^+^ decrease. (**A**) Under physiological conditions in young, healthy, non-obese people, most of the nicotinamide (NAM) generated in NAD^+^-consuming reactions is recycled to NAD^+^ in the salvage pathway due to low expression of NNMT and AOX1 in adipose tissue. (**B**) In the elderly, obese and people with diabetes, cardiovascular, pulmonary and renal diseases, highly expressed and activated NNMT and AOX1 exclude NAM from the salvage pathway. This “NAM drain” facilitates NAD^+^ depletion, particularly in stress conditions such as SARS-CoV-2 infection.

Oxidative stress, common in COVID-19, has the potential to up-regulate the expression of reactive oxygen species-inducible AOX1 [40,41]. Its activity may be detrimental for sustaining the NAD^+^ level in the setting of concomitant high NNMT expression. High expression of NNMT is inherent to conditions that predispose people for severe COVID-19. AOX1 in those conditions potentiates NNMT-mediated NAM clearance by removing MNAM-mediated inhibition of NNMT. Additionally, by generating 2PY and 4PY, AOX1 may stall the liberation of NAM in ADP-ribosylating reactions, thereby contributing to lower synthesis of NAD^+^ in the salvage pathway. Such “NAM drain” may aggravate, already existing, NAD^+^ deficiency in patients with predisposing conditions for severe COVID-19 (Figure 2).

## 3. The Role of NAD^+^ in Immune Response to SARS-CoV-2

Upon virus entry into a target cell, a variety of cellular pattern-recognition receptors recognize it and initiate the synthesis of interferons (IFNs). Interferons, secreted by virus-infected cells, are recognized by surrounding, uninfected, cells’ surface receptors to activate intracellular signaling pathways that induce the expression of interferon-stimulated genes (ISGs) [42]. Being essential for proper innate immune response, adequately activated IFNs restrain viral infection, while limiting tissue damage [43,44,45]. Accumulating evidence point to the contribution of deregulated innate immune response to the severe clinical presentation of COVID-19 [46,47]. The ability of SARS-CoV-2 to suppress type I IFN production and signaling facilitates its replication, dissemination and induction of an exuberant proinflammatory response [46,48,49,50,51]. Contrary to robust induction of type I IFN and IFNλ upon influenza virus infection, SARS-CoV-2 infection initially induces very weak IFN response, allowing its undisturbed replication [52]. Therefore, SARS-CoV-2 infection goes unnoticed for a prolonged period of time, causing no or very mild symptoms, contributing to a remarkable window of opportunity to spread among people. In a subset of people, however, sudden and severe symptoms develop, based on hyper-inflammatory response with subsequent impairment of gas-exchange function, leading to acute respiratory distress syndrome, multi-organ failure and death [53,54].

Upon SARS-CoV-2 infection, NAD^+^-consuming enzymes with antiviral properties: poly(ADP-ribose) polymerases (PARPs), sirtuins (SIRTs) and CD38 have prominent roles in immune response, ranging from the regulation of IFN induction, expression of interferon stimulated genes (ISGs) to the promotion of proinflammatory response [55,56,57]. Due to dependence of those enzymes on sufficient availability of NAD^+^ for their proper antiviral functions, NAD^+^ depletion and metabolites generated in the course of NAD^+^-consuming enzymes’ activity, profoundly shape the clinical outcome of SARS-CoV-2 infection.

### 3.1. PARPs in Immune Response to SARS-CoV-2

Post-translational modification ADP-ribosylation is catalyzed by members of the PARP family of enzymes (or more accurately termed ADP-ribosyltransferases, ARTs). In humans, 17 members of this enzyme family have been identified. According to their catalytic activity, they are classified as poly(ADP-ribose)polymerases, mono(ADP-ribose)transferases or inactive PARPs [58]. In the process of ADP-ribosylation, those enzymes degrade NAD^+^ to adenosine diphosphate ribose (ADPr) and liberate NAM, a form of vitamin B3, to be recycled into NAD^+^. In contrast to well-known multiple housekeeping roles of the best studied members, PARP1 and PARP2, which catalyze the formation of long, branched poly-ADP-ribose chains, roles of many other members of this enzyme family are much less well defined. Their emergence during vertebrate evolution suggests the involvement of several members of this enzyme family in host–pathogen interactions [59]. A number of PARP family members are ISGs, engaged in a variety of positive and negative feedback loops which finely tune antiviral IFN response [60]. Multiple roles of PARPs, and their NAD^+^-dependent activity, in response to SARS-CoV-2 infection can be envisioned based on the established mechanisms of action by which members of the PARP family exert their antiviral actions [60,61].

Viruses, on the other hand, employ multiple mechanisms to evade the host’s immune response [62].

Replication of coronaviruses (CoVs) is inhibited by ADP-ribosylation [63,64]. One of the mechanisms of SARS-CoV-2, and all other members of *Coronaviridae*, employed to counteract antiviral ADP-ribosylation is the removal of this post-translational modification [65,66,67]. A highly conserved macrodomain within nonstructural protein 3 (Nsp3) of SARS-CoV-2 is able to hydrolyze ADPr modifications [68]. Fehr et al., have demonstrated that the infection of mice with recombinant SARS-CoV harboring macrodomain mutations that reduce its de-ADP-ribosylation activity, leads to virus attenuation and a modest reduction in viral load in infected mice, despite normally replicating in cell culture. Although SARS-CoV macrodomain’s targets were not identified, the involvement of its de-ADP-ribosylating activity has been convincingly demonstrated in suppressing the early IFN and proinflammatory cytokine response [67].

Upon SARS-CoV-2 infection, PARP7, and PARPs 9–14 are upregulated [8]. Roles of those PARPs in antiviral response to SARS-CoV-2 are emerging, albeit their cellular or viral targets are still incompletely defined.

It has been experimentally established that the replication of SARS-CoV-2 is restricted by PARP13, also known as zinc finger antiviral protein (ZAP) [69]. The two most abundant ZAP isoforms, long (ZAP-L) and short (ZAP-S), both contain N-terminal RNA-binding domain and a central domain which binds poly(ADP)-ribose. ZAP-L isoform additionally contains an inactive C-terminal PARP domain with a cysteine (CaaX) motif which mediates S-farnesylation. This post-translational modification increases ZAP’s association with intracellular membranes [70,71]. Antiviral activity of ZAP is based on its binding to CpG dinucleotides in viral RNAs, leading to their degradation or inhibition of translation, making it the first line of defense against the virus.

The PARP domain with CaaX motif is required for ZAP’s interaction with several co-factors, including 3′-5′ exosome complex, TRIM25, KHNYN and OAS3-RNase L, involved in the restriction of viral replication [72]. In addition to being associated with intracellular membranes, ZAP is localized in stress granules, cytoplasmic structures lacking membrane, involved in the inhibition of translation under stress conditions, including viral infection [73]. In addition to PARP13, stress granules commonly contain PARPs 5a, 12, 14 and 15 as well as several ADP-ribosylated proteins. Poly ADP-ribose has been suggested to facilitate the accumulation of RNA-binding proteins in stress granules, thereby promoting their oligomerization [74]. Considering that polyADP-ribosylation is required for ZAP’s (PARP13) optimal antiviral activity, it remains to be established which PARP polyADP-ribosylates ZAP [75]. It has been reported that PARP14 monoADP-ribosylates ZAP at several amino acid residues and that this modification does not affect ZAP’s ability to bind RNA [76]. The consequences of PARP14-mediated monoADP-ribosylation of ZAP regarding its antiviral role remain to be established.

Russo et al., have established a cellular immunofluorescence-based assay able to detect ADP-ribosylation in response to treatment with viral RNA mimetic poly(I:C) or recombinant interferon gamma (IFNγ). They have demonstrated that ectopically expressed SARS-CoV-2 Nsp3 macrodomain can hydrolyze these modifications in human cells. IFN-induced ADP-ribosylation was detected predominantly in the cytosol in the form of punctate signal. It has been shown that this signal is dependent on IFN-stimulated expression of PARP9/DTX3L heterodimer [77]. Yang et al., have demonstrated that, on its own catalytically inactive, PARP9 mediates NAD^+^-dependent mono-ADP-ribosylation of ubiquitin (Ub) when in complex with DTX3L E3 ligase. ADP-ribosylation of carboxyl group of Ub Gly76 by PARP9/DTX3L, prevents ubiquitination. It has been shown, *in vitro*, that this inhibitory effect of ADP-ribosylation on ubiquitination is sensitive to NAD^+^ concentration [78]. Whether IFN-induced, PARP9/DTX3L-mediated ADP-ribosylation observed by Russo et al., targets Ub has to be investigated. It could be consequential for SARS-CoV-2-mediated inhibition of IFN signaling.

Recently, it has been reported that SARS-CoV-2 ORF7a usurps the host’s ubiquitin system to inhibit type I IFN response. SARS-CoV-2 ORF7a can be ubiquitinated by endogenous Ub. Among its seven Lys residues that may serve as ubiquitination sites, Lys119 has been identified as critical for ORF7a ubiquitination. Mutation of Lys119 residue to Ala that precludes SARS-CoV-2 ORF7a ubiquitination, significantly reduced inhibitory effect on IFN signaling, compared to wild-type (WT) ORF7a. Upon IFN-α treatment, WT ORF7a, in contrast to mutant K119A ORF7a, suppressed STAT2 (Signal transducer and activator of transcription 2) phosphorylation while having no effect on STAT1 phosphorylation. This is very important because transcription factors STAT1 and STAT2, in response to IFN, associate with IFN regulatory factor 9 (IRF9) to form a heterotrimeric transcription factor complex ISGF3 (Interferon-stimulated gene factor 3) [79]. Phosphorylated STAT1/STAT2 in the ISGF3 complex translocate to the nucleus and activate the transcription of ISGs. Ubiquitination of ORF7a has been shown to hinder STAT2 phosphorylation, thereby inhibiting nuclear translocation of STAT1 and IFN-α signaling. Consequently, WT ORF7a ubiquitination diminished the transcription of several ISGs, including 2′-5′-oligoadenylate synthetase 1 (OAS1) [80].

In the study by Russo et al., treatment with viral RNA mimetic poly(I:C) or recombinant IFNγ induced the expression of PARP9 and DTX3L, increased the level of ADP-ribosylation and STAT1 phosphorylation, together with a robust increase in OAS1 transcription. However, knockout of PARP9 or DTX3L did not decrease STAT1 phosphorylation nor did it affect the induction of OAS1. In this experimental system, poly(I:C) and recombinant IFNγ were able to induce IFN-stimulated expression of PARP9 and DTX3L with a consequent increase in ADP-ribosylation. The negative effect of ADP-ribosylation on ubiquitination-mediated SARS-CoV-2 ORF7a antagonism of IFN response, could not occur due to the absence of SARS-CoV-2 ORF7a and possibly other viral proteins which antagonize IFN response [80]. Consistent with the lack of ORF7a which uses ubiquitination to hinder STAT2 phosphorylation and transcription of ISGs, in the experimental system of Russo et al., PARP9/DTX3L-mediated ADP-ribosylation or its reversal by SARS-CoV-2 Nsp3 macrodomain had no effect on the transcription of ISGs [77]. The experimental evaluation of the possibility that PARP9/DTX3L-mediated ADP-ribosylation hinders SARS-CoV-2 ORF7a ubiquitination-mediated evasion of IFN response is needed. Considering the sensitivity of PARP9/DTX3L activity to cellular NAD^+^ level (*K_M_* of Dtx3L/PARP9 for NAD^+^ is 197 ± 64 µM), it may be an important factor which determines the initial successful restriction of viral replication [78].

The importance of the abovementioned ISG OAS1 in restricting SARS-CoV-2 replication has been reported recently [81]. It recognizes short stretches of double-stranded viral RNA and activates RNaseL, leading to its degradation. It has been shown that OAS1 binds two conserved stem loops in the SARS-CoV-2 5′ untranslated region. Two major isoforms of OAS1 (p46 and p42), in humans, are generated by alternative splicing. Only a longer, prenylated, p46 isoform of OAS1 is able to efficiently detect SARS-CoV-2. Albeit all human genotypes encode the last exon present in the transcript encoding p46, single nucleotide polymorphism (SNP) in intronic region determines its usage [81]. Despite individual genetic predisposition for expressing p46, it is conceivable that SARS-CoV-2 ORF7a may hinder IFN-stimulated transcription of OAS1 and, despite the ability of the host to generate p46, diminish its antiviral effect. Dependent on cellular NAD^+^ level, PARP9/DTX3L may or may not preclude SARS-CoV-2 ORF7a-mediated inhibition of STAT2 phosphorylation, reliant on ubiquitination.

The ability of SARS-CoV-2 Nsp3 macrodomain, which binds ADP-ribose more tightly (K_d_ = 10 µM) than SARS-CoV macrodomain (K_d_ = 24 µM), to reverse ADP-ribosylation implies the possibility that viral replication would be limited only when the extent of ADP-ribosylation exceeds the capacity of viruses to reverse it [82]. Since the availability of NAD^+^ is a limiting factor for PARPs’ activity, its level is critical for their antiviral effects.

Production of IFN, in response to CoV infection or poly(I:C) treatment, has been shown to depend on ADP-ribosylation. More specifically, Grunewald et al., have demonstrated that PARP14-mediated ADP-ribosylation has an important role in inducing type I IFN, although other PARPs are likely to participate in IFN induction since pan-PARP inhibitor 3-aminobenzamide (3-AB) had a more pronounced negative effect on IFN production than a selective PARP14 inhibitor (compound 8K) [64]. After triggering an initial type I IFN response, SARS-CoV-2 downregulates type I IFN expression in infected cells, leading to delayed type I IFN responses [83,84]. Thus, it is conceivable that the strength of initial SARS-CoV-2-induced activation of PARPs, gauged by the availability of NAD^+^, might determine the extent of type I IFN induction and SARS-CoV-2 replication.

Inadequate early type I IFN responses, due to insufficient availability of NAD^+^, would permit viral replication and trigger more exuberant immune response [85].

### 3.2. Sirtuins and COVID-19

Sirtuins, originally identified as histone lysine deacetylases are now known to act upon numerous nonhistone substrates, thereby regulating their function and affecting diverse cellular functions. There are seven members of this enzyme family in humans, localized to distinct subcellular compartments (SIRT1, 6 and 7-nucleus; SIRT3, 4 and 5-mitochondria; SIRT1 and 2-cytoplasm) [86,87]. They have a prominent role in maintaining cellular homeostasis through the regulation of energy status and stress responses [88]. Immune response to viral infection and ensuing inflammation represent considerable stress which triggers vigorous stress response. The dependency of SIRTs’ activity on sufficient availability of NAD^+^ is evident from their high Michaelis–Menten constant for NAD^+^, compared to other NAD^+^-consuming enzymes [89]. Due to their relatively high requirement for NAD^+^, SIRTs are sensitive to fluctuations in the cellular NAD^+^ level.

Sirtuins regulate transcription and metabolism, thereby affecting numerous cellular pathways central to both viral life cycle and antiviral response. They affect the expression of both host and viral genes through targeting many important transcription factors such as p53, nuclear factor kappa B (NFκB) and forkhead box O1 (FOXO1) [90,91,92].

The activity of SIRTs is gauged by the availability of NAD^+^, thus it is closely linked to cellular metabolic status. In addition to being reliant on cellular metabolism, SIRTs directly regulate various metabolic pathways [93].

Cellular metabolism has an important role in regulating both innate and adaptive immune responses. SIRTs participate, at multiple levels, in the regulation of metabolic shifts that occur after immune stimulation and in the resolving phase of the response [94]. Knockdown of individual SIRTs has been reported to increase the yield of human cytomegalovirus (HCMV), herpes simplex virus (HSV-1), human adenovirus 5 (Ad5) and influenza virus H1N1 in human cells. Those results imply that SIRTs hinder the replication of viruses regardless of their biology (DNA vs. RNA, enveloped vs. naked, rapidly vs. slowly replicating viruses). The mechanisms of SIRTs’ antiviral effect on different viruses are expected to be diverse, since the effect of different SIRTs’ silencing varied among four viruses explored [95].

There are very limited data on SIRTs’ involvement in COVID-19. Yao et al., analyzed the transcriptome of peripheral blood mononuclear cells (PBMCs) obtained from healthy subjects and COVID-19 patients with moderate disease, acute respiratory distress syndrome (ARDS) and those recovering from ARDS by single-cell RNA sequencing and found suppressed signaling mediated by SIRTs in severe COVID-19 patients [96]. Bordoni et al., have recently shown that PBMCs from COVID-19 patients have a high expression of p53 and significantly decreased expression of SIRT1, together with elevated levels of proinflammatory cytokines IL-1β, TNF-α, IL-8, and IL-6. Concomitant with upregulation of p53 and downregulation of SIRT1, COVID-19 patients had decreased expression of key genes for lymphocyte homeostasis IL-7R and B cell linker (BLNK), compared to healthy donors. Those changes were accompanied with a significant increase in apoptotic B and T cells and reduction in IgK and IgL chains in lymphopenic COVID-19 patients [97].

Those results support the hypothesis put forward by Miller et al. [98], that in NAD^+^-deficient COVID-19 patients, due to either advanced age, obesity or type 2 diabetes mellitus (T2DM), SIRT1 activity is compromised and unable to downregulate ADAM17 (a disintegrin and metalloproteinase domain 17, also known as TNF-α-converting enzyme) and restrict uncontrolled increase in TNF-α and IL-6. To be systemically active, TNF-α and the cytokine receptors, IL-6R and TNF-R, must be cleaved by proteinase ADAM17 [99]. An endogenous inhibitor of ADAM17, tissue inhibitor of metalloproteinase 3 (TIMP-3), is positively associated with SIRT1. It has been shown that the overexpression of SIRT1, in smooth muscle cells, increases TIMP-3 promoter activity, while SIRT1 inhibition or downregulation reduces its expression [100]. When not inhibited by TIMP-3, due to low SIRT1 activity-mediated TIMP-3 reduction, ADAM17 causes the release of TNF-α and IL6 and contributes to inflammation and possibly to the development of an uncontrolled hyperinflammatory response in COVID-19 [98,101].

### 3.3. CD38 in COVID-19

CD38 is a multifunctional protein acting as a signaling receptor and an enzyme. It is predominantly expressed in immune cells upon stimulation by inflammatory cytokines, endotoxins and interferon [102,103,104]. It exists in a transmembrane and a soluble form [105]. In the cellular membrane, CD38 can be oriented with its carboxyl-terminal, catalytic domain facing the extracellular environment (type II orientation) or facing the inside of the cell (type III orientation) [106].

Using NAD^+^ as a substrate, CD38 mainly acts as a NAD^+^-glycohydrolase to generate ADPr or as a cyclase to generate a small amount of cyclic ADPr (cADPr) [107]. Cyclic ADPr is converted by CD38 hydrolase activity into ADPr. The majority of CD38 has type II orientation and acts as an ecto-NADase [108]. Considering the presence of a much higher intracellular level of NAD^+^ and intracellular activity of cADPr as a second messenger signaling molecule, extracellular orientation of the CD38 catalytic domain posed a topological paradox [109]. In addition to using NAD^+^ as a substrate, CD38 metabolizes extracellular NAD^+^ precursors, nicotinamide mononucleotide (NMN) and nicotinamide riboside (NR), thereby limiting their availability for importation into cells and NAD^+^ synthesis [110]. This role of type II membrane bound CD38 in limiting precursors for NAD^+^ synthesis calls into question the accuracy of a long-believed existence of topological paradox [111].

Preferentially at acidic pH, CD38 catalyzes a base-exchange reaction that replaces the nicotinamide group of NADP^+^ with nicotinic acid (NA) to generate nicotinic acid adenine dinucleotide phosphate (NAADP) and hydrolyze NAADP to adenosine diphosphate ribose phosphate (ADPrP) [112,113]. All products of the CD38 catalyzed reactions are Ca^2+^-mobilizing second messengers, implicating its role in intracellular calcium homeostasis [114].

Additionally, CD38 is involved in the regulation of extracellular adenosine (ADO), a nucleoside involved in the control of inflammation and immune responses. Through its nicotinamide adenine dinucleotidase (NADase) activity, CD38 generates cADPr, ADPr and NAM. Nucleotide pyrophosphatase/phosphodiesterase (NPP) CD203a transforms ADPr into adenosine monophosphate (AMP), which is further converted into ADO and inorganic pyrophosphate (PPi) by 5′-nucleotidase (5′-NT) CD73 [115]. Production of ADO and its ligation to A2A adenosine receptor (A2AR) inhibits cytolytic activities of effector T lymphocytes. In the setting of anti-pathogen immune response, this effect is beneficial as it restrains excessive tissue damage. Moreover, A2AR stimulation by ADO significantly increases the number of regulatory T cells (Treg), leading to longer lasting T cell inhibitory effect [116].

Additionally, the resolution phase of acute inflammation is regulated by CD38-generated Ca^2+^-mobilizing second messengers cADPr and NAADP, which increase the expression of tristetraprolin (TTP, known also as ZFP36). In a feed-back loop, TTP regulates CD38 at a post-transcriptional level by decreasing *Cd38* mRNA stability. Downregulation of CD38 increases NAD^+^ level needed for SIRT1 activity. Deacetylation of TTP by SIRT1 potentiates its activity during the onset of resolution phase, leading to the degradation of proinflammatory transcripts including tumor necrosis factor alpha (*TNFA*) [117].

Given the multifunctional nature of CD38 and its crucial role in regulating the NAD^+^ level, the production of Ca^2+^-mobilizing second messengers and ADO, it is conceivable that the contribution of CD38 to COVID-19 severity is immense, as extensively reviewed by Horenstein et al. [57].

Being a major culprit in NAD^+^ decline during aging and other conditions associated with chronic low-grade inflammation, such as obesity and metabolic disorders, CD38 represents an attractive therapeutic target. However, its mandatory role in efficient immune response to pathogens, and not less important role in resolution phase of inflammatory response, should be considered when contemplating targeting CD38 to treat viral diseases. Tightly regulated activity of CD38 is important for homeostasis, since complete absence of its activity, as demonstrated in CD38 knockout mice, causes impairment in insulin secretion, increases susceptibility to bacterial infections and leads to changes in social behavior [118,119,120]. Central to the maintenance of physiological functions is the robustness of the NAD^+^ metabolism, which deteriorates with aging and in various pathologies.

Covarrubias et al., recently reported the accumulation of proinflammatory M1-like macrophages, expressing high levels of CD38, in visceral adipose tissue and the liver of mice during aging and in acute response to inflammation. Senescent cells, in aging tissues, secrete an array of inflammatory cytokines (known as senescence associated secretory phenotype, SASP) which induce macrophages to proliferate and express CD38. High CD38-mediated NADase activity of macrophages in adipose tissue and the liver during aging and in response to lipopolysacharide (LPS)-induced acute inflammation contributes to the NAD^+^ level decline [121].

The involvement of NNMT and AOX was suspected to contribute to NAD^+^ decline, upon LPS-induced expression of CD38, and enhanced liberation of NAM in the course of its NADase activity. The validity of the hypothesis that upon exuberant liberation of NAM by CD38 in inflammatory conditions, its methylation by NNMT and subsequent oxidation of MNAM to 2PY and 4PY, by AOX, excluding NAM from the NAD^+^ salvage pathway, has been interrogated. The level of NAD^+^ and its metabolites were determined in adipose tissue and the liver of WT and CD38 knockout mice upon treatment with LPS. Only in WT mice, levels of MNAM and its metabolites increased in both adipose tissue and the liver, concomitant with LPS-induced expression of CD38, in contrast to CD38 knockout mice. Those results support the proposition that the main route of excess NAM clearance under inflammatory conditions is its NNMT-mediated methylation. Methylation of NAM in the liver may profoundly influence the NAD^+^ level in peripheral tissues in which NAM is the major precursor for NAD^+^ synthesis [121]. Importantly, in addition to their role in the NAD^+^ metabolism, NAM and MNAM have opposing immunomodulatory properties. High levels of NAM have been demonstrated to reduce the production of inflammatory cytokines in macrophages [122]. Recently, Kilgour et al., have found that MNAM accumulates in ovarian tumor-infiltrating T cells, which do not express NNMT, in contrast to fibroblasts and tumor cells. The uptake of MNAM, produced by neighboring cells, induces T cells to secrete proinflammatory cytokine TNFα [123]. The contribution of MNAM, generated during antiviral immune response-related inflammation, to the secretion of TNFα by T cells remains to be established.

## 4. Direct Contribution of Reduced NAD^+^ Level to a Range of COVID-19 Pellagra-like Symptoms

NAD^+^ deficiency, regardless of its etiology, contributes to the development of various symptoms resembling pellagra, a “disease of the 4 Ds” (for dermatitis, diarrhea, dementia and death) [18]. Sub-clinical pellagra is often not recognized due to lack of routinely used specific tests for diagnosing this type of deficiency. It causes a wide range of symptoms and often not all characteristic symptoms are present, thus *forme fruste* of pellagra commonly goes unrecognized. Pellagra, caused by dietary vitamin B3 and tryptophan deficiency is rare in the developed world. It sporadically occurs upon chronic alcohol abuse and in people with malabsorption states [124,125,126]. Regardless of sufficient dietary intake of its precursors, NAD^+^ deficiency may occur when NAD^+^ consumption is not adequately counterbalanced by its biosynthesis due to “NAM drain”, mediated by NNMT and AOX1 activity. Endogenously caused NAD^+^ deficiency is common to old age and pathological states predisposing for development of severe COVID-19 [17,121].

Despite the fact that NAD^+^ depletion in response to the SARS-CoV-2 infection is not a unique phenomenon, several characteristics of this virus may precipitate exuberant NAD^+^ depletion. SARS-CoV-2 has the ability to disguise its presence and delay, evade and suppress the immune system, allowing its undisturbed replication early on upon infection. Considering that SARS-CoV-2 Nsp3 macrodomain avidly degrades ADPr set by antiviral PARPs, it is conceivable that NAD^+^ consumption and liberation of NAM are highly elevated. The robustness of the host’s NAD^+^ metabolism to respond to this stressful event shapes the clinical presentation of COVID-19.

A range of symptoms reminiscent of pellagra have been reported, even in patients with mild or moderate COVID-19, supporting the notion of a robust, initial NAD^+^ depletion in response to SARS-CoV-2 infection.

### 4.1. Mucocutaneous and Gastrointestinal Manifestations

The name of niacin deficiency, pellagra, is coined from Italian words *pelle*—skin and *agra*—rough, since skin manifestations are the most noticeable signs of this disease. Tissues most affected by niacin deficiency, and ensuing NAD^+^ deficiency, are those with the highest cell turn-over rates such as the skin and gastrointestinal tract [18].

Numerous reports have been published on the occurrence of various skin manifestations and gastrointestinal disturbances in COVID-19 [127,128,129]. COVID toes and COVID tongue are among presentations frequently observed during the COVID-19 pandemic [130,131,132]. Interestingly, in the majority of the patients presenting with those symptoms, SARS-CoV-2 has not been detected either by RT-PCR or serological tests. It has been suggested that chilblain-like manifestations on fingers and toes likely represent a consequence of a vigorous innate immune response to the virus and its clearance before developing specific antibodies. Chilblains have been observed in rare monogenic type 1 interferonopathies, in which an antiviral innate immune response is excessively activated [133]. Nevertheless, such vigorous innate immune response can cause transient NAD^+^ depletion, manifesting in acral skin lesions characteristic for pellagra [134]. Likewise, COVID tongue resembles changes in the tongue, described in pellagra, ranging from geographic type lesions to general atrophy of the lingual papillae or marginal atrophy of the tongue [131,135].

### 4.2. Smell, Taste and Nociception Disturbances Related to Deficient Oxytocin Secretion

Early on during the SARS-CoV-2 pandemic, the loss of smell and taste has been described as one of the common characteristic symptoms of COVID-19 [136,137,138]. Green has described in 1971., based on clinical experience, that those symptoms are associated with subclinical pellagra. Indeed, treatment with niacin resolved those sensory disturbances in his patients [139]. At that time, the mechanism by which niacin deficiency causes those symptoms was not known. Since then, it has been established that oxytocin (OT) signaling is involved in human social behavior, including olfactory perception [140].

Oxytocin levels depend on CD38 expression and activity since its secretion involves release of Ca^2+^ from ryanodine-sensitive stores, dependent on cADPr and NAADP. Those two Ca^2+^-mobilizing signaling molecules exert both autocrine and paracrine action. Expression of CD38 is not restricted to neurons of the supraoptic and paraventricular nuclei, where OT is mostly synthesized for both systemic and central release. Rather, cADPr and NAADP may be synthesized outside of those cells and taken up by OT-producing neurons [120,141]. In addition to being involved in olfactory perception, OT, although not produced locally in taste buds, elicits Ca^2+^ signals through OT receptors in murine taste buds [142]. Therefore, it is conceivable that acute NAD^+^ depletion upon SARS-CoV-2 infection contributes to inefficient CD38-dependent OT secretion, inducing smell and taste perception disturbances in COVID-19 patients.

The use of OT as adjuvant therapy for this disease has been proposed on the basis of its anti-inflammatory and immune-modulating properties [143,144,145,146]. However, to the best of my knowledge, endogenous OT levels in COVID-19 patients have not been assessed, most likely due to the highly invasive nature of obtaining samples of cerebrospinal fluid and controversial reports on the correlation of OT levels measured in plasma or saliva with those from cerebrospinal fluid [147,148].

The reports on age and sex differences in the plasma level of OT in humans are scarce. In male mice, the systemic OT level declines with age. Elabd et al., have reported three-fold lower OT levels in aged mice (18 to 24 months old) compared to young (2 to 4 months old) mice [149]. Regarding sex differences in human plasma OT levels, reported findings are inconsistent. Weisman et al., reported higher plasma OT levels in men than women, while Plasencia et al., found a higher plasma OT level in women [150,151]. The disparate results on sex differences regarding the plasma OT level, in those two studies, most likely stem from sampling women at different phases of the menstrual cycle and inclusion or exclusion of breastfeeding subjects [150,151].

An important insight into the involvement of the NAD^+^ level in the regulation of OT secretion comes from a report that elevating NAD^+^ levels by NR supplementation corrects OT levels in CD157 KO mice, deficient in cADPr formation. Both CD157 and CD38 are involved in calcium-dependent production of OT. Those enzymes produce cADPr, calcium-mobilizing second messenger, using NAD^+^ as a substrate. Increased NAD^+^ levels, upon NR supplementation, allowed higher CD38 activity, corrected cADPr deficit and restored OT production in CD157 KO mice [152].

Decreased NAD^+^/NADH ratio is characteristic biochemical aberration in diabetic tissues [153]. In line with the mandatory requirement of sufficient NAD^+^ levels for calcium signaling-dependent secretion of OT, decreased serum OT levels have been reported in type II diabetes mellitus patients and obese people when compared to subjects with normal glucose tolerance and normal weight [154]. Patients suffering from T2DM have been reported to have impaired taste and smell recognition more frequently than healthy subjects, albeit the relationship with OT levels has not been interrogated [155,156,157,158]. Whether the occurrence of olfactory and gustatory dysfunctions in COVID-19 patients relate to decreased OT levels, related to decreased NAD^+^ levels and an impairment in calcium signaling, remains to be investigated.

Among its multiple actions, OT is involved in the suppression of inflammatory pain nociception. It has been reported that OT attenuates pain through the pain-sensing transient receptor potential vanilloid subtype 1 (TRPV1). The proposed mechanism of OT-mediated attenuation of pain sensing is initial activation of TRPV1, which is followed by its pronounced desensitization [159]. In addition to nociception, TRPV1 is involved in cough hypersensitivity. It has been suggested that, based on functional magnetic resonance imaging studies showing that the same brain areas are activated by pain and cough, post-COVID-19 hypersensitive cough and musculoskeletal pain may be related to common pathways of central hypersensitivity [160]. Whether a decreased level of OT is contributing to the development of this hypersensitivity needs to be determined.

### 4.3. Insulin Secretion Impairment Due to Deficient CD38-Mediated Ca^2+^ Mobilization

In addition to being involved in OT secretion, CD38-mediated Ca^2+^ mobilization is indispensable for insulin secretion, as shown in a model of CD38 KO mice. Those mice had lower serum insulin levels and impaired glucose tolerance, compared to the control mice. This impairment was rescued by beta cell-specific expression of CD38 [118]. Okamoto proposed a model in which NAD^+^ depletion, by its excessive consumption upon PARP activation, aimed to repair DNA damage caused by oxygen radicals or the alkylation of DNA, inhibits insulin synthesis and secretion, ultimately leading to beta cell death [161]. Those early studies on the involvement of CD38 activity in insulin secretion were centered on cADPr as the main Ca^2+^ signaling molecule in this process [118,162,163]. Subsequently, it has been demonstrated that although the cADPr level increases upon glucose stimulation, it is not sufficient for insulin secretion [164]. Johnson and Misler have demonstrated that initiation of insulin signaling depends on NAADP, another CD38-derived molecule [165]. Heister et al., recently proposed that glucose triggers initial NAADP-dependent Ca^2+^ mobilization from likely acidic stores, prior to the influx of Ca^2+^ and insulin exocytosis [166].

The dependence of glucose-stimulated insulin secretion on NAD^+^ has been demonstrated 15 years ago by Revollo et al., in a study investigating the role of extracellular Nampt (eNampt), which has previously been considered to act as a cytokine named pre-B cell colony-enhancing factor (PBEF) or an insulin-mimetic hormone visfatin. It has been demonstrated that it has a robust NAD^+^ biosynthetic activity and that its haplodeficiency or chemical inhibition reduces NAD^+^ biosynthesis and glucose-stimulated insulin secretion in pancreatic islets *in vitro* and *in vivo*. Supplementation with NMN, a product of the Nampt reaction, corrected this effect of Nampt inhibition, demonstrating that Nampt-mediated NAD^+^ biosynthesis is critical for beta cell function [167]. In mammals, NAMPT exists in two forms, intracellular (iNAMPT) and extracellular (eNAMPT). The lack of a signal sequence for secretion implied the possibility that eNAMPT presence outside the cell stems from cell lysis [168]. It has since been established that SIRT1-mediated deactylation of iNAMPT, in adipocytes, predisposes it for secretion. Extracellular NAMPT is secreted from differentiated adipocytes and has a 2–4-fold higher enzymatic activity than iNAMPT [167].

Considering the dependence of beta cell function on NAD^+^, its overwhelming depletion and liberation of NAM, in response to SARS-CoV-2 infection, can profoundly influence insulin secretion and glucose tolerance. Indeed, from the early days of the pandemic, reports were arising on the ability of SARS-CoV-2 to induce hyperglycemia in people without previous diagnosis of diabetes. The infection-induced hyperglycemia is not unique for SARS-CoV-2 as it occurs with other infections [169,170]. The mechanism/s by which SARS-CoV-2 infection induces hyperglycemia and occasionally reported new onset of diabetes are currently uncertain. There is no clear proof of direct SARS-CoV-2-induced damage to beta cells [170,171]. Only the long-term follow-up will resolve whether the SARS-CoV-2 infection precipitates the onset of permanent diabetes to a greater extent than other infections. Hyperglycemia, associated with community-acquired pneumonia, occurs frequently in patients without pre-existing diabetes, but can promote the onset of permanent diabetes, particularly in older people [169].

As the NAD^+^ level decreases with aging, together with the diminishment of the ability to respond to infection-induced NAD^+^ depletion by replenishing it in the salvage pathway, it may have a more permanent effect on beta cell function in older people [172]. Whether other conditions associated with the reduced fitness of the NAD^+^ metabolism predispose COVID-19 patients to permanent impairment of beta cell function remains to be investigated.

## 5. Effects of the “NAM Drain”, Other Than NAD^+^ Deficiency, in COVID-19

**Figure 3:**
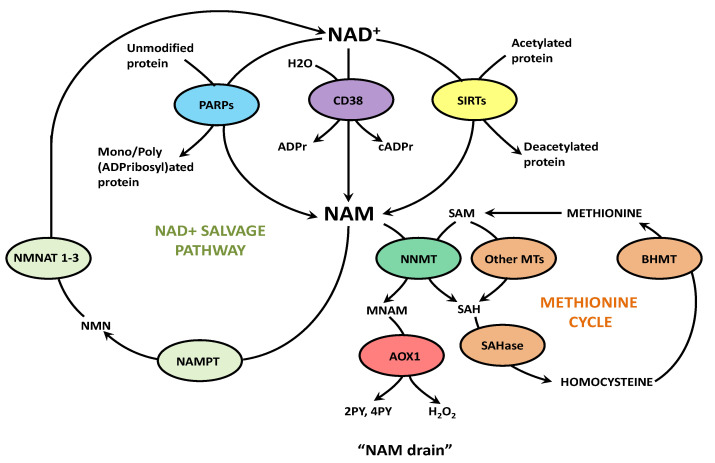
“NAM drain” concomitantly affects NAD^+^ salvage pathway and methionine cycle. Excessive nicotinamide (NAM) clearance by its nicotinamide N-methyltransferase (NNMT)-mediated methylation excludes NAM from the salvage pathway and consumes methyl units from S-adenosyl methionine (SAM) to generate N1-methylnicotinamide and S-adenosyl homocysteine (SAH). Enzyme S-adenosyl-L-homocysteine hydrolase (SAHase) converts SAH into homocysteine (Hcy). Conditions of high NAD^+^ consumption and liberation of NAM, as well as excessive dietary intake of vitamin B3, promote “NAM drain”-mediated clearance of surplus NAM. This process stalls NAD^+^ synthesis in the salvage pathway, decreases methylation potential of the cell and augments the generation of (Hcy).

Several symptoms of COVID-19, dissimilar to primary pellagra, are not related to NAD^+^ deficiency itself, but rather to disturbances caused by the process of its evolution—“NAM drain”. Removal of excessive NAM, liberated in NAD^+^-consuming reactions, is achieved by its NNMT-mediated methylation. This reaction consumes methyl units from S-adenosyl methionine (SAM) to generate MNAM and S-adenosylhomocysteine (SAH) [173]. S-adenosyl-L-homocysteine hydrolase (SAHase), subsequently, converts SAH to adenosine and homocysteine (Hcy) (Figure 3) [174].

High expression and activity of NNMT in adipose tissue, is the major source of Hcy, contributing to the onset of hyperhomocysteinemia, a risk factor for atherosclerosis. Importantly, the release of Hcy, from mouse liver and white adipose tissue samples, has been shown to increase more than 46% upon incubating cells in serum-free media containing 1 mM NAM for 15 h. Conversely, NNMT’s product and inhibitor, MNAM, potently decreased the Hcy level [175]. In a multicenter, retrospective study of 313 patients hospitalized for COVID-19, the plasma Hcy level was found to be significantly higher in non-survivors [176]. In another study, involving 273 COVID-19 patients, hyperhomocysteinemia has been found predictive of computed tomography (CT) imaging progression in lungs [177]. This rise in Hcy is indicative of enhanced consumption of methyl units from SAM by methyltransferases. Ulanovskaya et al., has proposed that NNMT represents a “methylation sink” in cancer and compromises the methyl donor balance [173].

High activity of NNMT in the liver, by reducing methylation capacity, reduces methylation of the connective tissue growth factor (*CTGF*) gene promoter with a consequent increase in its expression. This upregulation of CTGF promotes the development of liver fibrosis [178]. As CTGF also has a crucial role in the development of lung fibrosis, NNMT-mediated decline in methylation may potentially facilitate the progression of lung fibrosis observed in COVID-19 patients [179].

Apart from influencing methylation capacity, NNMT has recently been reported to have a protective role in endothelial cells exposed to oxidative stress. Inhibition of its activity by 5-amino-1-methylquinoline (5MQ) and 6-methoxynicotinamide (JBSF-88), in human endothelial HMEC-1 and HAEC cells, decreased their viability upon menadione-induced oxidative stress. The ratio of phosphorylated (Ser 47) SIRT1 to total SIRT1 increased both in response to NNMT inhibitors and menadione-induced oxidative stress. The authors proposed that NNMT exerts its protective function against oxidative stress through SIRT1 activity [180]. This decrease in total SIRT1 protein expression, upon NNMT inhibition, is in line with the report on SIRT1 protein stabilization upon increasing MNAM levels or Nnmt expression in the liver [181]. Phosphorylation of SIRT1 at Ser47, by c-Jun N-terminal kinase 1 (JNK1), induces its brief activation, followed by ubiquitination and degradation in proteasome [182]. It is conceivable that in the course of COVID-19 progression and stalling of NAD^+^ flux due to acute NAD^+^ depletion, less NAM will be liberated and processed by NNMT, leading to SIRT1 degradation and activation of KP. Likewise, in primary pellagra the excretion of NAM and its metabolites is low and KP is upregulated, as judged from an increase in the KYN/Trp ratio [183].

Transcription of *ACMSD*, coding for the enzyme whose activity diverts Trp towards ATP production in TCA cycle, away from KP, is cooperatively regulated by PGC1α and HNF4α [23]. SIRT1 regulates the activity of PGC-1α by deacetylating it in a NAD^+^-dependent manner [184]. Hence, either low expression of SIRT1, due to its compromised stability, or its depressed activity, due to NAD^+^ deficiency, may result in decreased ACMSD expression and activation of KP.

COVID-19-mediated NAD^+^ depletion, alike primary pellagra, leads to the activation of KP, lymphopenia and elevation of inflammatory markers. High activity of KP in COVID-19, with consequent lowering of Trp and accumulation of neuroactive metabolite QA, may account for some of the neurologic manifestations in COVID-19 [12]. The pathophysiological basis of COVID-19-associated neurological symptoms are not well elucidated. Despite speculation that SARS-CoV-2 may enter the central nervous system, SARS-CoV-2 has not been readily detected by RT-PCR in CSF of COVID-19 patients. The evidence from CSF and brain tissue of deceased COVID-19 patients analyses point to the immune activation and inflammation as primary drivers of neurological manifestations. Some of the symptoms, such as fatigue, impaired concentration, depression, headache and sensory disturbances, may persist after the clearing of the infection for a period of time [185]. Even patients who experienced mild to moderate COVID-19 during acute infection, occasionally report persisting symptoms not related to virus-induced organ damage. Those symptoms, commonly referred to as “long COVID-19”, often resemble myalgic encephalomyelitis/chronic fatigue syndrome (ME/CFS). “Long COVID-19” and ME/CFS share characteristic changes related to redox imbalance, inflammation, an impaired ability to generate adenosine triphosphate and general hypometabolic state. Some of those derangements are consistent with changes related to insufficient robustness of the host’s NAD^+^ metabolism to buffer infection-induced stress and regain homeostatic state upon resolution of the infection. Changes common to both “Long COVID-19” and ME/CFS are an increase in Hcy, impairment of the NAD^+^ salvage pathway, increased KYN/Trp ratio and quinolinic acid, consistent with the upregulation of incompletely executed KP [186].

Similar to malnutrition-induced pellagra in endogenously caused NAD^+^ deficiency, the clinical presentation varies according to its severity.

The persistence of NAD^+^ deficiency upon resolution of infection will likely depend on the initial robustness of the NAD^+^ metabolome.

## 6. Concluding Remarks and Open Questions

The strength of the initial immune response to SARS-CoV-2, to restrain its replication, depends on the cellular NAD^+^ level.

High potency of SARS-CoV-2 in reversing antiviral ADP-ribosylation confers higher resistance of this virus to the restriction of replication and permits the development of serious disease in patients with pre-existing, subclinical NAD^+^ deficiency.

Endogenously caused NAD^+^ deficiency, common in old age and other predisposing conditions for development of serious COVID-19, most likely stems from elevated NAD^+^ degradation in NAD^+^-consuming reactions, coupled with NAD^+^ salvage pathway stalling, mediated by cooperative activity of NNMT and AOX1 [17,21,35,121].

This deficiency can be detected by determining the ratio between the NAD^+^ level in blood and the NAM metabolite’s level in urine [153].

Despite many similarities in clinical consequences of dietary and endogenous NAM deficiency, the latter cannot be remedied by vitamin B3 supplementation alone. Inhibition of AOX1 may alleviate the disturbance of the NAD^+^ salvage pathway, and together with adequate nutrition contribute to the restoration of the cellular NAD^+^ level. In support of the proposition that AOX1 inhibition may increase the NAD^+^ level comes from the results of screening 304 active compounds, covering a broad range of oncology targets, to evaluate their effect on the NAD^+^ level in A549 lung cancer cells. Raloxifene hydrochloride caused a two-fold increase in NAD^+^/NADH levels. The EC_50_ value for NAD^+^/NADH was 700 nM at 24 h and decreased to 30 nM after 46 h of treatment [187]. Raloxifene, a prescription drug for the treatment of osteoporosis, is a potent AOX1 inhibitor in clinically relevant doses [188]. However, the use of raloxifene for treatment of COVID-19 outside of a controlled clinical trial is not recommended.

Administration of high doses of vitamin B3 alone is not likely to prevent SARS-CoV-2-aggravated NAD^+^ deficiency, and may even be harmful.

Considering the important physiological roles of NAD^+^-consuming enzymes, an attempt to elevate NAD^+^ level by their inhibition may be detrimental, particularly in the face of ongoing infection.

Although NAD^+^ depletion is not a unique phenomenon of the SARS-CoV-2 infection, this pandemic brought to light metabolic vulnerability inherent in an aging population and people suffering from an array of non-communicable diseases, now well established as risk factors for developing serious COVID-19. By the virtue of its high capacity to actively reverse antiviral ADP-ribosylation, this virus accentuated pre-existing states of NAD^+^ deficiency, not related to insufficient dietary intake of its precursors, but characteristic for the states of diminished NAD^+^ salvage pathway functionality due to excessive “NAM drain”.

Detailed characterization of this process would provide a foundation for developing modalities aimed to prevent and alleviate NAD^+^ deficiency not only related to COVID-19, but to a whole spectrum of “inflammaging”-related conditions which have emerged during this pandemic as risk factors for worse clinical outcome.

## Data Availability

Not applicable.
